# ‘Cashing in’ nicotine pouches for prizes

**DOI:** 10.1136/tc-2024-058691

**Published:** 2024-06-15

**Authors:** Page D Dobbs, Grace Kong, Micah L Berman, Lisa Henriksen

**Affiliations:** 1 University of Arkansas Fayetteville, Fayetteville, Arkansas, USA; 2 Psychiatry, Yale School of Medicine, New Haven, Connecticut, USA; 3 College of Public Health, The Ohio State University, Columbus, Ohio, USA; 4 Moritz College of Law, The Ohio State University, Columbus, Ohio, USA; 5 Cancer Control Program, James Comprehensive Cancer Center, The Ohio State University, Columbus, Ohio, USA; 6 Stanford Prevention Research Center, Stanford University School of Medicine, Palo Alto, California, USA

Non-tobacco oral nicotine products (eg, flavoured nicotine pouches, non-therapeutic nicotine gums, lozenges, tablets and nicotine gummies) are the second most popular nicotine product among US youth behind electronic cigarettes (e-cigarettes).^1-4^ Such popularity corresponds with increased sales in the USA.^5-8^ Popular brands of nicotine pouches include Zyn (Philip Morris International), On! (Altria) and Velo (British American Tobacco).^5 9^ These products are sold in a variety of flavours that may appeal to young audiences.^12^ In addition, nicotine pouches are marketed on radio, television, mobile apps and online displays^1 2 9^ using various themes such as circumventing tobacco-free and smoke-free indoor air laws and depictions of young people engaging in leisure activities.^5^ Given the importance of understanding the role of marketing in attracting new users^10-13^ and regulatory implications of such practice, we examined a social media platform commonly used by young people (ie, TikTok) to identify marketing and promotion strategies for flavoured nicotine pouches.

In the 2009 Tobacco Control Act, the US Congress instructed the US Food and Drug Administration (FDA) to reissue a 1996 regulation prohibiting tobacco companies from giving away any non-tobacco item (using the brand logo or otherwise) with the purchase of cigarettes or smokeless tobacco.^14^ However, the 2016 Deeming Rule failed to extend this marketing restriction to newly deemed tobacco products, including non-tobacco oral nicotine pouches that do not meet the legal definition of smokeless tobacco. Further, a 2012 Sixth Circuit Court of Appeals decision (Discount Tobacco City & Lottery v. USA) concluded that the FDA’s ban on continuity programmes—reward programmes that give items or other gifts to repeat customers—violated the tobacco companies’ First Amendment rights.^15^ The court concluded that because continuity programmes, such as frequent flyer programmes, ‘are designed to maintain the loyalty of existing customers, not to attract new ones,’ the FDA could not use its concern about youth uptake to limit such programmes. Thus, the FDA (without further rulemaking) cannot prohibit the use of continuity programmes, and its ban on other forms of distributing non-tobacco items does not apply to nicotine pouches that do not contain tobacco leaf.

Given this policy loophole, tobacco companies are recycling past marketing strategies, such as reward programmes, with emerging products including non-tobacco oral nicotine pouches. TikTok, a social media platform popular among young audiences,^16^ displays videos of people explaining that Zyn has a reward programme where users can scan a QR code on the back of the can for 15 points. Points can be ‘cashed in’ on the Zyn website for prizes as large as brand name grills (figure 1). A review of Zyn, On! and Velo’s official websites demonstrated that all three brands featured a rewards programme, with advertised prizes including Uber gift cards (ie, On! and Zyn) and ‘fun activities and merchandise’ (ie, Velo) (see figure 2). Some of these rewards programmes also promoted large point values for recruiting others to register on their website, which was similar to the gamification strategies used by the 2020 Camel Crush sweepstakes that gave prizes to participants who registered on their website but no purchase or payment was required.^17^ Although Zyn’s fine print explains that users can only redeem 60 containers per month, these continuity programmes encourage users to increase their chances of winning by buying more of their product and encouraging others to engage with the brand.

TikTok videos showed young women collecting and ‘cashing in’ Zyn containers. While some videos claimed the products were used by their boyfriends or friends, others displayed women using pouches or bragging about how their nicotine addiction got them an espresso machine. Thus, loyalty programmes may attract new female customers seeking to receive rewards. Such evidence undermines the industry’s argument that these programmes are solely used to establish brand loyalty among adult users by demonstrating an effort to grow their market among young females. Further, several non-tobacco oral nicotine pouch companies are owned by large tobacco companies that can send users coupons and discounts for a variety of tobacco products, which has been associated with increased tobacco initiation among new users and lower successful cessation among current users.^18^ Thus, continuity programmes for non-tobacco oral nicotine pouches have the potential to attract new users of tobacco products rather than just establishing brand loyalty.

Oral nicotine products are regulated differently in different countries (ranging from no regulations to the prohibition of sales),^8^ and they are not yet regulated as strictly in the US as other tobacco products^19^; however, the recent increases in use of these products among US youth warrant regulatory attention due to the harm nicotine can have on brain development.^1-4 20^ Aggressive marketing strategies by these non-tobacco oral nicotine pouch companies on social media that lack proper regulation and are used frequently by young people suggest vulnerability among price-sensitive populations that may be enticed to win prizes they could not normally afford.^4^ Such groups are likely to include youth and young adults who are at risk of nicotine initiation and continued use, including the nearly 3 million youth in the US who report past-30 day tobacco use.^21^ To prevent young people from initiating the use of non-tobacco oral nicotine products, the FDA is encouraged to close regulatory gaps in the Deeming Act by expanding marketing restrictions to newer tobacco products. The FDA could revise the Deeming Rule to better regulate the marketing of non-tobacco oral nicotine pouches (and other emerging products), and it could try again to regulate continuity programmes, given that the 2012 court decision was based on the evidence available at the time. Considering the marketing practices highlighted here and trend data about increasing youth and young adult use of non-tobacco oral nicotine pouches,^2-4^ the FDA should assess what it can do to close the regulatory gaps that tobacco companies are exploiting.

## Figures and Tables

**Figure 1:**
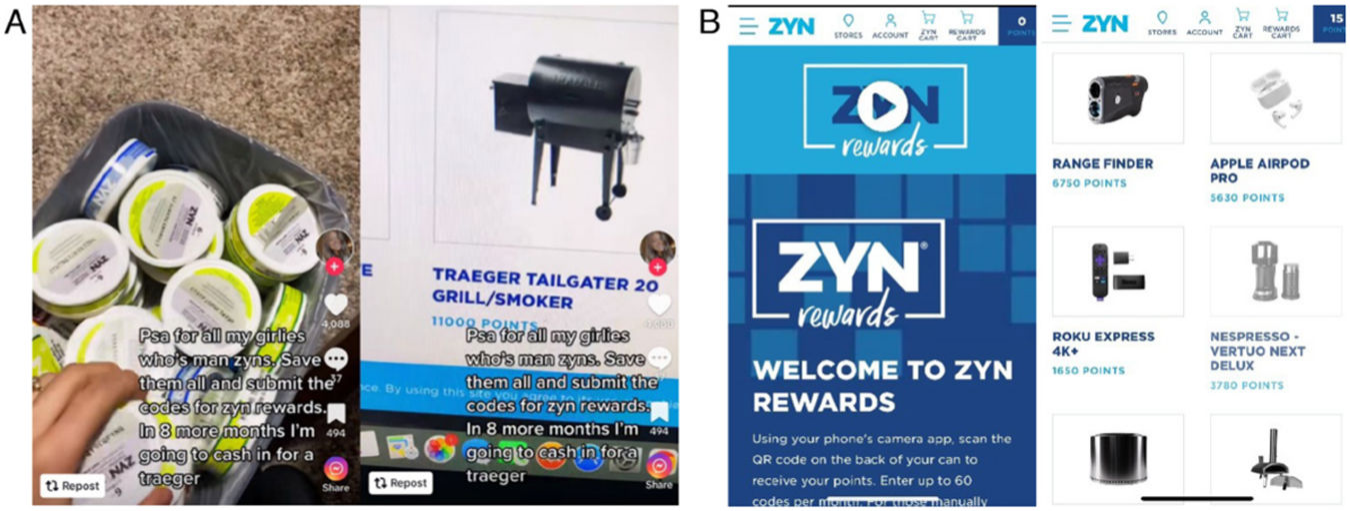
(A) Zyn rewards advertised on TikTok. (B) Zyn rewards website.

**Figure 2:**
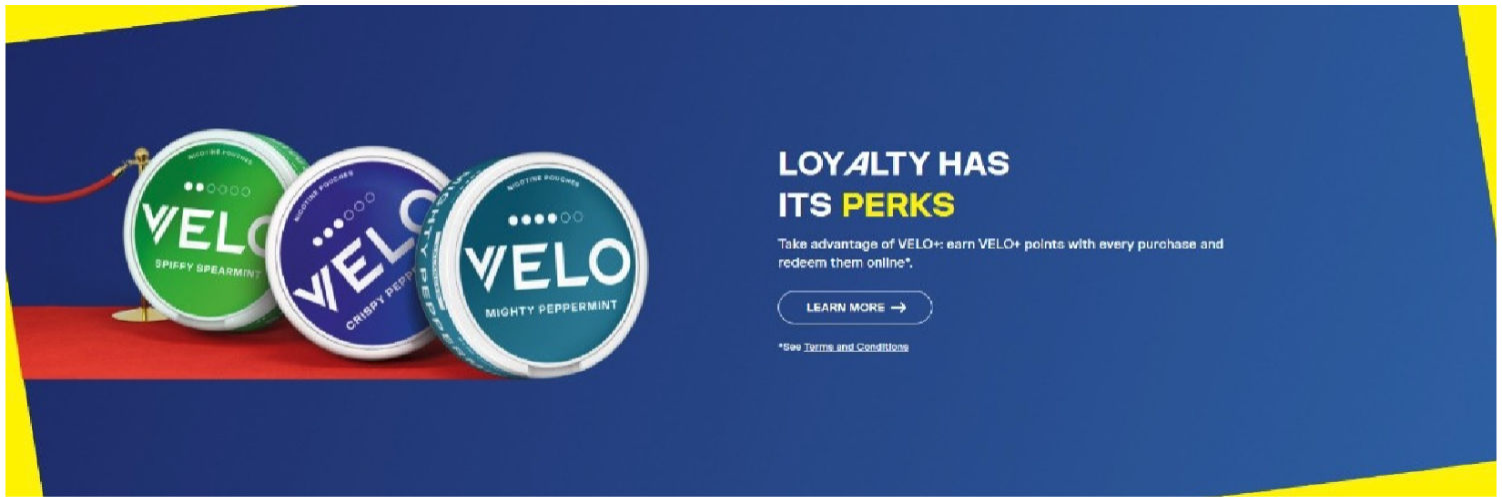
Velo reward advertised on their website.
